# Somatosensory and Gustatory Profiling in the Orofacial Region

**DOI:** 10.3390/diagnostics12123198

**Published:** 2022-12-16

**Authors:** Amely Hartmann, Claudia Welte-Jzyk, Irene Schmidtmann, Christian Geber, Bilal Al-Nawas, Monika Daubländer

**Affiliations:** 1 Private Practice for Oral and Maxillofacial Surgery, Echterdinger Straße 7, 70794 Filderstadt, Germany; 2 Department of Oral and Maxillofacial Surgery, University Medical Centre of the Johannes Gutenberg University of Mainz, 55131 Mainz, Germany; 3 Orthodontics Outpatient Clinics, University Medical Centre of the Johannes Gutenberg University of Mainz, 55131 Mainz, Germany; 4 Department of Neurology, University Medical Center of the Johannes Gutenberg University of Mainz, 55131 Mainz, Germany; 5 Institute of Medical Biostatistics, Epidemiology and Informatics (IMBEI), University Medical Centre of the Johannes Gutenberg University of Mainz, 55131 Mainz, Germany; 6 DRK Schmerz-Zentrum, 55131 Mainz, Germany; 7 The German National Institute for State Examinations in Medicine, Pharmacy and Psychotherapy, IMPP, 55116 Mainz, Germany

**Keywords:** quantitative sensory testing, standardized testing battery, orofacial pain, neurophysiological changes, sensory function, neurosensory disturbances, COVID-19, gustatory testing

## Abstract

Quantitative sensory testing (QST) is a standard procedure in medicine to describe sensory patterns in various pathologies. The aim of this prospective clinical study was to define reference values of the trigeminal nerve (V3), including taste qualities, to create a compatibility for sensory loss or gain in pathologies. Fifty-one patients were included, and a standardized testing battery with 11 QST parameters according to the German Research Network on Neuropathic Pain (DFNS) was applied complemented by quantitative gustatory assessments. Significant somatosensory differences were found between the test sites (MDT at the chin, WDT at the lower lip) but no effect was detected for gender, age, and between body types. Taste sensitivity was dependent on concentration, gender (females being more sensitive) and increasing age (for bitter and sour taste). We provide reference values for somatosensory and gustatory testing of the facial area. Our data facilitate the detection of neurosensory abnormalities in the orofacial region. This might also serve as a control setting for COVID-19.

## 1. Introduction

The facial area is heavily innervated by nerves. Due to surgical interventions or pathologies, neurophysiological disturbances can occur [1]. The underlying somatosensory patterns can be described by means of Quantitative Sensory Testing (QST), implemented by the German Research Association for Neuropathic Pain (DFNS) in 2006 [2]. Evaluation of the QST thermal and mechanical parameters can display the sensory abnormalities of thin unmyelinated Aδ, C-fibers and thick myelinated Aß-fibers. The documented sensory plus and minus signs indicate possible central or peripheral sensitization [2,3,4,5,6,7].

The consecutive precise presentation of somatosensory profiles can make a significant contribution to the understanding of pathophysiological mechanism. A mechanism-based classification of the individual pain syndromes will help to generate new therapy options [2,8]. To assess sensory deficits, it is necessary to create a normative database with age and gender-specific reference values. These data already exist for some areas of the body, like hand and foot [2,4,9,10]. However, although data for the face exist [9,11], data that provide a comprehensive range of QST normative data in the innervation area of the trigeminal nerve (V3), especially intraorally, are pending.

Therefore, the purpose of this prospective clinical study was to obtain QST reference data in four areas innervated by the trigeminal nerve (lower lip, tongue, chin, and gingiva) and to complement them with gustatory tests. Aspects such as the influence of age, gender, and test site (extra-/intraorally) were considered.

## 2. Materials and Methods

### 2.1. Study Design

This study was conducted according to the ethical principles of the World Medical Association 2013, Declaration of Helsinki. Informed and written consent was obtained by each person voluntarily taking part in the study. Using a prospective human study design, ethical approval was given from the local ethics committee (ethics committee of Rhineland-Palatinate, 837.168.13 (8853)). This study followed the appropriate EQUATOR guidelines.

Only healthy subjects (aged 18–70) were enrolled. They were recruited via notices placed in the Johannes Gutenberg University Mainz. Subjects of different sexes and age groups were included. Exclusion criteria were systemic and underlying local diseases. If previous orofacial injuries, diabetes, neurological or psychiatric disorders were documented, subjects were not considered. Smokers and patients with chronic pain states were excluded as well. 

### 2.2. Materials

#### 2.2.1. Materials for QST

Instruments used were slightly modified according to our previous reports [1,12,13].

For thermal testing, Modular Sensory Analyzer (MSA) Thermotester from SENSELab/Somedic^®^ (Sweden) was used with a 9 × 9 mm thermode, including a Peltier element and cooling water supply. To avoid infections, the thermode was covered with a saliva-proof film. 

Mechanical detection threshold (MDT) was evaluated by using von-Frey-filaments (OptiHair2-Set^®^, Marstock Nervtest, Germany) with intensities of 0.05, 0.08, 0.125, 0.25, 0.5, 1, 2, 4, 8, 16, 32, 64, 128, 256 and 512 mN. The contact surface of the glass filaments is spherical and has a diameter of 0.5 mm.

Mechanical pain threshold (MPT), mechanical pain sensitivity (MPS) and wind-up ratio (WUR) were investigated with needle stimulators (Pinprick, MRC Systems GmbH, Germany) with intensities of 8, 16, 32, 64 and 128 mN. The contact surface of the pin pricks is flat and 0.25 mm in diameter.

A possible mechanical dynamic allodynia was analyzed using a cotton ball (3 mN), a cotton swab (100 mN) and a SENSELab^®^ Brush-05 recorded by Somedic (200–400 mN). Because of a lack of disinfection ability, a disposable toothbrush is used intraorally. 

#### 2.2.2. Materials for Taste Measurement

Taste qualities ‘sweet, sour, salty and bitter’ were evaluated using test strips from Burghardt Messtechnik (Wedel, Germany) in ascending concentration. Contact area is 2 cm and is coated with sucrose for ´sweet´ (0.4, 0.2, 0.1, 0.05 g/mL Sucrose), citric acid for ‘sour’ (0.3, 0.165, 0.09, 0.05 g/mL citric acid), sodium chloride for ‘salty’ (0.25, 0.1, 0.04, 0.016 g/mL sodium chloride) and quinine hydrochloride for ‘bitter’ (0.006, 0.0024, 0.0009, 0.0004 g/mL quinine hydrochloride).

### 2.3. Methods

#### 2.3.1. Preliminary Examinations

**Figure 1:**
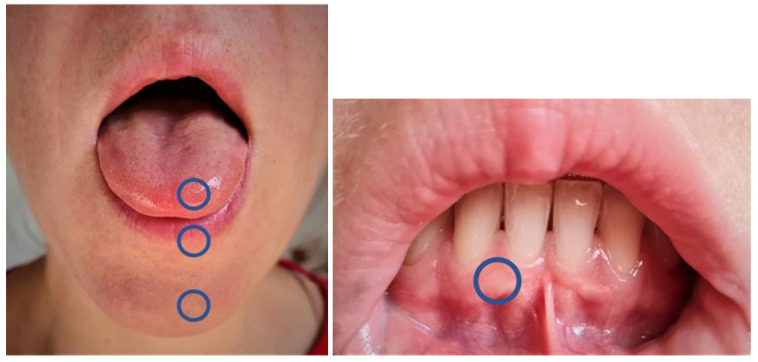
Quantitative Sensory Testing (QST) measurement points (V3, trigeminal nerve). Left front third of the tongue, lower lip, chin and right fixed gingiva.

Measurements were performed according to the German Research Association for Neuropathic Pain (DFNS). The standardized testing protocol of the quantitative sensory testing to a standardized testing battery [8,9] is a psychophysiological investigation method, consisting of 7 tests with 13 parameters: detection thresholds for warm and cold (WDT, CDT), paradoxical heat sensations (PHS) generated by alternating cold and warm stimuli (thermal sensory limen, TSL), pain thresholds for thermal stimuli (CPT, HPT), mechanical detection thresholds for vibration and contact (VDT, MDT), mechanical pain thresholds (MPT), mechanical pain sensitivity (MPS) and mechanical dynamic allodynia (DMA), pain summation (WUR) and pain thresholds for pressure (PPT). For anatomical reasons, evaluation of pressure pain threshold (PPT) and vibration threshold (VDT) was not possible and consecutively, pressure algometer and tuning fork were not used intraorally. To guarantee a standardized testing procedure in a reproducible setting, the investigators were trained with written instructions [14,15,16]. In this study, preliminary examinations were performed to train the examiner to intra-individual measurement fluctuations and to define suitable measurement areas for QST data collection. The tests were carried out on the lower lip, as well as on the front third of the tongue. Further test sites were at the fixed gingiva and chin (Figure 1). Each measurement was performed bilaterally. Examination took about one hour. 

#### 2.3.2. Thermal Testing

Thermal testing enabled the determination of perception and pain thresholds for cold, warm, and hot stimuli, therefore checking the functionality of C- and Aδ-fibers. The thermode of the computerized thermal tester (see ‘Materials’) offered a baseline temperature of 32 °C. The temperature changed with a speed of 1 °C/to a maximum of 50 °C and a minimum of 5 °C. If the subject signaled to feel the stimulus by pressing a stop button, it was switched off and then returned to its starting temperature of 32 °C. This happened also automatically at maximum and minimum temperature to prevent thermal irritation of the skin.

The healthy volunteers were asked to close their eyes during the test-procedure. They were not able to see the test monitor. 

The following parameters were determined using the ‘method of limits’ [14,15,16]:

##### Cold Detection Threshold (CDT)

The thermode was set to the respective measuring area, and after an initial run of 6 s to enable temperature adaptation, it was cooled continuously at a rate of 1 °C/s. The patient was asked to press the stop button as soon as she/he perceived cooling. This process was repeated twice to gain an arithmetic mean of the three data. 

##### Warm Detection Threshold (WDT) 

After an initial run of 6 s, heat was raised with a speed changed by 1 °C/s. The subject was advised to press the stop button by recognizing warming up of the thermode. The process was carried out three times and WDT defined as the arithmetic mean of these measurements.

##### Thermal Sensory Limen (TSL) and Paradoxical Heat Sensation (PHS)

For the detection of TSL, the thermode started from the base temperature of 32 °C, and it was alternately heated and cooled. During this temperature change, however, the thermode did not return to baseline temperature. Each modulation in temperature perceived by the subjects was indicated by pressing the stop button. Temperature modulation was performed six times, so that the arithmetic mean of three temperature differences calculated the thermal difference threshold. Cold stimuli perceived by the subjects as hot or warm were identified as paradoxical heat sensations.

##### Cold Pain Threshold (CPT)

The thermode was cooled down analogous to testing of the CDT but subjects were instructed to press the stop button only by perceiving cold as “burning”, “pricking”, “drilling” or “pulling”. These unpleasant sensations did not have to be accompanied by extreme pain. After defining the threshold, the thermode returned to baseline temperature. This process was carried out three times, defining an arithmetic mean. 

##### Heat Pain Threshold (HPT)

As performed in the previous test, the test person was instructed to use the stop button to indicate an unpleasant sensation (“burning”, “piercing”, “drilling”, “pulling”) after heat increase. HPT was defined as the arithmetic mean of three threshold values.

#### 2.3.3. Mechanical Testing

##### Mechanical Detection Threshold (MDT) 

Whilst performing this test, functionality of Aß-fibers was checked. MDT was performed using the ‘method of levels’, according to Rolke et al. [4]. This method consists of five explicit over and five subliminal stimuli. Von-Frey-filaments had a skin contact for about 2 s, building a S-shape. Starting with the intensity of 16 mN, the von-Frey-filaments were placed in descending intensity, until the subject no longer felt any stimulus. Afterwards, ascending order was performed. This process was repeated four times, determining a geometric mean for a total of ten values. Subject were instructed to close their eyes during the entire testing procedure. 

##### Mechanical Pain Threshold (MPT) 

This test could present neurosensory pathways indicating fiber function of the Aδ- and C fibers. By using pinprick needle stimulators with an intensity of 8, 16, 32, 64, and 128 mN, a defined force with a defined weight was applied to the skin. The flat circular surface was placed for 1–2 s in a perpendicular angle to the examination area and a tube slides out. The principle applied was the ‘method of levels’ again; in this case, the supra-threshold was determined because of ascending order of the pinpricks (starting with the lowest intensity of 8 mN). The test person was asked to define the stimuli either as ‘sharp’ or ‘blunt’. ‘Blunt’ was defined as a stimulus perceived like a touch. The first stimulus perceived as ‘sharp’ was noted as the first supra-threshold value. Based on this value, pinpricks in decreasing order were used afterwards for the first value defined as ‘blunt’. After defining five supra and five subliminal stimuli, the geometric threshold was calculated.

##### Mechanical Pain Sensitivity (MPS) and Dynamic Mechanical Allodynia (DMA)

The set was used (Pinpricks in the same intensities; cotton ball, Q-tip and brush with a force of 200–400 mN, or toothbrush, as mentioned above) in a defined order; the test person was asked to rate painfulness (rating scale 0 to 100) according to the applied stimuli. Simple perception of touch was rated as “0” and the maximum imaginable pain defined as “100”. Any sharp, stabbing or burning sensation should be rated with a number above 0. The standardized test included the application of 35 needle and 15 light touch stimuli. Stimuli were applied with an interval of approximately 10 s to prevent a possible wind-up phenomenon. MPS was defined as the geometric mean of needle stimuli. DMA was described as the mean value of touching sensations.

##### Wind-Up Ratio (WUR)

Only one pinprick with an intensity of 64 mN was used. First, the subject rated a single needle stimulus analogous to the previous test. Needle stimuli should be rated with a number above “0”. The pinprick was applied with a frequency of 1 s, ten times on the same skin area (total area 1 cm^2^). Afterwards, painfulness of the series of stimuli was assessed using the rating scale. After repeating the series five times, WUR was calculated as the quotient of the sum of painful testing batteries divided by the sum of the painfulness of individual stimuli.

#### 2.3.4. Principle of Taste Testing

The coated contact area of the test strips was placed on the left and right side of the tongue in a defined order. Testing procedure started with the lowest concentration of each taste quality, followed by an increasing order. In total, 32 taste qualities of different concentrations were considered. The subjects were instructed not to lick at the taste strips or to close the mouth, and to check only taste buds in the front third of the tongue. After each application, the test person was asked to briefly moisten the tongue to create the same conditions for each testing. The test was performed based on a standardized protocol and answers documented by an evaluation sheet.

### 2.4. Statistical Analysis

#### 2.4.1. QST Data

QST data were collected using an EXCEL datasheet (Microsoft, USA). Data were handled as described by Rolke 2006. The statistical analysis was performed using IBM^®^ SPSS^®^ Statistics version 23.0 for Windows^®^. CDT, WDT, TSL, MDT, MPT, MPS, WUR data were log transformed for normal distribution. QST parameters CPT and HPT were processed without any transformation. Data were compared using paired *t*-tests (side to side comparison). 

To identify the effect of the areas a two-factor analysis of variance (ANOVA) was applied. For age and gender specific influences, a two-factor analysis of variance (ANOVA) was applied as well. *p*-values < 0.05 were described as significant.

#### 2.4.2. Evaluation of Taste Data

Data were coded in a binary form (0 = tasted incorrectly or not detected, 1 = tasted in a correct way) using EXCEL (Microsoft, Redmond, WA, USA). Statistical analyses were performed using SAS (SAS 9.3 Software Institute Inc., Cary, NC, USA). GLIMMIX procedure was used to describe the influence of the parameters: concentration, age, tongue side and quality of taste on the ability to taste of each subject. A generalized linear mixed model was adjusted, representing a binary-logistic regression but taking multiple—notPiggnecessarily independent—measurements per person into account. *p*-values of < 0.05 were defined as significant. 

## 3. Results

### 3.1. Patients

51 patients were included in this study (31 women, mean age 40.4 ± 15.0 and 20 men with a mean age of 44.5 ± 15.1). QST parameters were evaluated bilaterally. MDT was not evaluated in 4 patients (MDT, *n* = 47). 

### 3.2. QST

**Figure 2:**
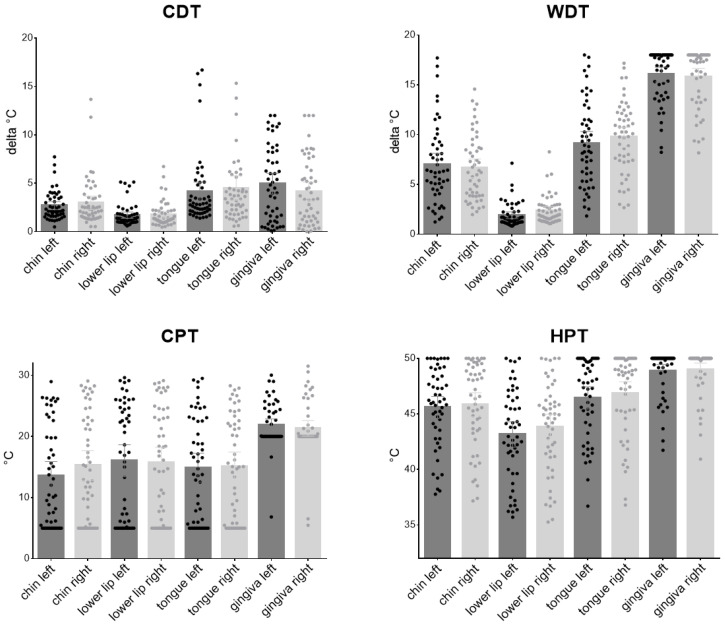
Thermal thresholds (detection and pain raw values) for extraoral (chin, lower lip) and intraoral (tongue tip, gingiva) left and right areas. (CDT = Cold Detection Threshold, CPT = Cold Pain Threshold, WDT = Warm Detection Threshold, HPT = Heat Pain Threshold).

**Figure 3:**
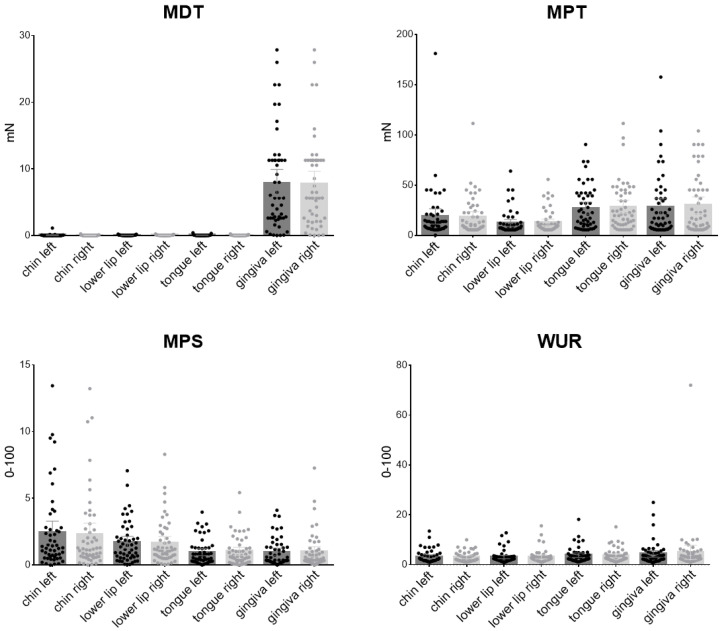
Mechanical thresholds (detection and pain raw values) for extraoral (chin, lower lip) and intraoral (tongue tip, gingiva) left and right areas. (MDT = Mechanical Detection Threshold, MPS = Mechanical Pain Sensitivity, MPT = Mechanical Pain Threshold, WUR = Wind Up Ration).

QST values was assessed bilaterally in four different orofacial areas, two extraoral (chin, lower lip) and two intraoral (tongue tip, gingiva) resulting in eight test sites (Figure 2 and Figure 3).

#### 3.2.1. Thermal Thresholds

Thermal stimuli were perceived earlier in the extraoral than the intraoral region with the lower lip being the most sensitive (Figure 2 CDT, WDT) resulting in lower detection thresholds: for CDT it was −1.7± 1.15 °C on the left and −1.77 ± 1.27 °C on the right side, for the left chin CDT, cold was recognized after cooling by 2.68 ± 1.48 °C and for the right chin after cooling by 2.98 ± 2.45 °C. Detection of cold at the intraoral regions, where only after cooling down by 4.10 ± 3.66 °C for the left and 4.51 ± 4.47 °C for the right tongue tip and 4.96 ± 3.83 °C for the left and 4.12 ± 3.46 °C for the right gingiva, warm detection was later in the intraoral regions (gingiva left 16.03 ± 2.65 °C and right 15.83 ± 2.92 °C, tongue tip left 9.13 ± 4.22 °C and right 9.74 ± 3.59 °C) than in the extraoral areas (chin left 7.01 ± 3.97 °C and right 6.63 ± 3.3 °C, lower lip left 1.90 ± 1.2 °C and right 2.38 ± 1.41 °C), with again the lip as the most sensitive area. For the thermal pain thresholds, cold pain was detected similar at the chin (left 13.54 ± 8.29 °C, right 15.26 ± 8.65 °C), the lower lip (left 16.02 ± 9.37 °C, right 15.71 ± 8.89 °C) and the tongue tip (left 14.89 ± 8.36 °C, right 15.08 ± 8.35 °C), whereas for the gingiva (left 21.81 ± 3.78 °C, right 21.32 ± 4.54 °C), the cold pain threshold lower. Similar results were found for HPT (chin left 45.59 ± 3.52 °C, chin right 45.82 ± 3.86 °C, lower lip left 43.15 ± 4.09 °C, lower lip right 43.83 ± 4.05 °C, tongue tip left 46.43 ± 3.55, tongue tip right 46.84 ± 3.69 °C) except that the gingiva was the most pain insensitive to heat stimuli (left 48.84 ± 2.11 °C, gingiva right 48.99 ± 2.09 °C). PHS did not significantly occur in healthy subjects.

#### 3.2.2. Mechanical Thresholds

Detection of light mechanical stimuli (MDT) was achieved for light stimuli for the chin (left 0.08 mN ± 0.16, right 0.05 mN ± 0.04), the lower lip (left 0.08 mN ± 0.04, right 0.08 mN ± 0.05) and the tongue tip (left 0.08 mN ± 0.07, right 0.07 mN ± 0.04), but for more intense stimuli for the gingiva (left 7.89 mN ± 7.21, right 7.76 mN ± 6.68). Detection of sharp versus blunt stimuli (MPT) was easier for the extraoral regions (chin left 18.82 mN ± 26.6 and right 18.41 mN ± 18.96, lower lip left 12.50 mN ± 12.19 and right 13.02 mN ± 11.56) than for the intraoral areas (tongue tip left 27.15 mN ± 21.34 and right 28.36 mN ± 23.88, gingiva left 28.19 mN ± 30.77 and right 30.10 mN ± 28.91). In line with the results found for MPT, pain rating to sharp mechanical stimuli is higher in the extraoral areas (chin left 2.47 ± 2.94 and right 2.32 ± 2.91, lower lip left 1.71 ± 1.55 and right 1.66 ± 1.67, tongue tip 0.98 ± 0.95 and right 1.04 ± 1.10, gingiva left 0.98 ± 1.04 and right 0.99 ± 1.36). DMA did not significantly occur in healthy subjects. WUR was not different for all regions q (chin left 3.23 ±2.64 and right 3 ± 2.01, lower lip left 3.09 ± 2.53 and right 3.10 ± 2.79, tongue tip left 3.82 ± 3.12 and right 3.68 ± 2.73, gingiva left 4.34 ± 4.58 and right 4.99 ± 9.98). 

According to Rolke et al. 2006 for the cheek, we considered the values by age (<40, >40) and gender (Table 1). The data for the masseter we considered in a like manner. 

There were no significant side effects detectable, except for WDT at the lower lip (<0.01) and MDT at the chin (<0.5) (Table 2), which is why the data from both sides were thus combined.

Mean values of QST parameters did significantly differ between the four test sites (chin, lower lip, tongue tip and gingiva (CDT *p* < 0.001, WDT *p* < 0.001, TSL *p* < 0.001, HPT *p* < 0.001, MDT *p* < 0.001, MPT *p* < 0.001, MPS *p* < 0.01) (Table 3). Concerning gender, no significant effect was shown on QST values, but for cold detection, we found an effect due to age (*p* < 0.001), which is influenced by gender (age ∗ sex *p* = 0.003) (Table 3). For WDT, we found r WDT the difference in the position is affected by age (position ∗ age *p* = 0.0002).

### 3.3. Taste

All 51 patients were able to distinguish between the taste qualities of sweet, sour, salty and bitter in four different concentrations on the right and left tongue side. 

**Figure 4:**
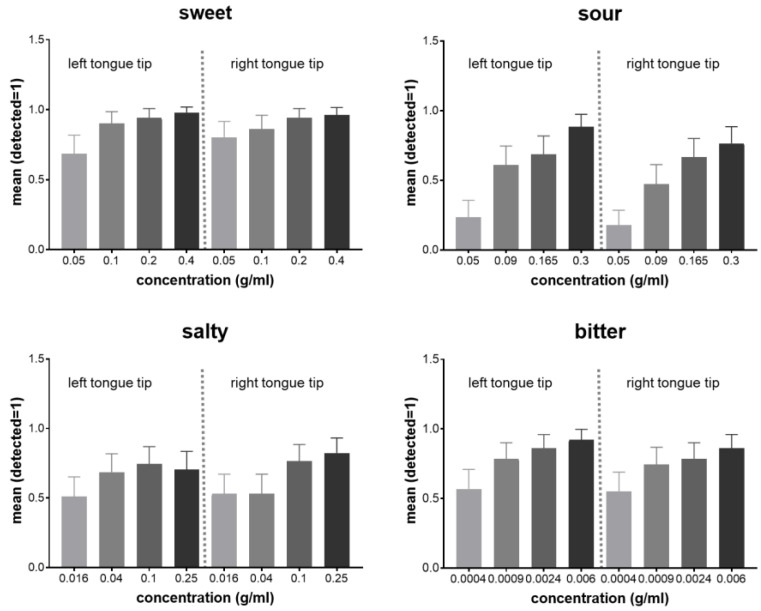
Taste qualities in different concentrations on the left and right tongue side.

A highly significant effect of the concentration on taste ability was found for all taste qualities (sweet *p* < 0.0001, sour *p* < 0.0001, salty *p* = 0.0002 and bitter *p* < 0.0001). Taste ability increased with higher concentrations (Figure 4). Women proved to have more taste sensitivity (sour *p* = 0.011, salty *p* < 0.001 and bitter *p* = 0.008). For the taste qualities bitter (*p* = 0.015) and sour (*p* = 0.002), a significant effect of age was detected with diminishing taste ability with increasing age (>40). 

## 4. Discussion

### 4.1. QST

An adequate diagnostic method is required to objectively determine functional deficiencies and to achieve an adequate therapy [14]. Therefore, QST aids to deliver prognostic measurements of pathologies. Small and large nerve fiber functions can be precisely described, revealing sensory loss and gain indicating small and large fiber neuropathies [2,15]. Together with psychological aspects, QST can be a predictor of chronic pain states [16]. To define pathogenetic changes, which can be iatrogenic or idiopathic, it is mandatory to refer to standard/norm values.

In previous studies using QST for somatosensory profiling of patients with neuropathic pain conditions [8,17], it could be shown that specific patterns of sensory loss and gain are independent of the underlying etiology of the nerval damage such as post-herpetic neuralgia or diabetes. Three different somatosensory phenotypes have been identified: predominant sensory loss (cluster 1), thermal hyperalgesia (cluster 2, equivalent to ‘peripheral sensitization’) and mechanical hyperalgesia (cluster 3, equivalent to ‘central sensitization’). As these patterns are linked to different pathomechanisms of neuropathic pain [8], it should be possible to individually stratify the antineuropathic treatment. So far, this has been shown in proof-of-concept studies. Therefore, beyond its diagnostic value, QST offers the potential for more personalized therapy of pain syndromes. 

For the orofacial region, reference values are documented for V2 of the trigeminal nerve and intraorally [18,19,20]. The lack of data for V3 and the need to define reference values is pointed out in other studies [11]. Therefore, the aim of this study was to establish a reproducible protocol with reference values of the mucosa and skin of the facial area (V3 of the trigeminal nerve). By defining reference values at four orofacial V3 areas (chin, lower lip, tongue tip and gingiva) in healthy subjects, future studies could refer to these data indicating various pathogenic processes such as chronic pain states of the face (lesions of the trigeminal nerve). According to our best knowledge, this is the first study describing the whole somatosensory profile for V3 in subjects, including gustatory qualities. This study was able to prove the efficacy and reliability of this diagnostic intra- and extraoral tool. Reference values could be established representing the baseline for further studies in neurophysiological changes. The evaluated data could potentially be used for future z-scores and a reference to a defined standard neurophysiology of V3 in the facial innervation. In pathologies, the value of z transformed QST data (the established norm values defining the “0” baseline) will illustrate individual profiles [1,4]. 

Comparing both sides of the face, no side effects were found as expected, except for WDT at the lower lip and MDT at the chin. Rolke et al. [4] found no significant right-to-left difference for any of the QST parameters (correlation coefficients left-right between 0.78 and 0.97). In other studies, the same was evaluated [19,20]. It can be observed that for all QST values there was a wide scattering of the individual values, indicating a large interindividual variation. It is noticeable that warm and cold stimuli were perceived more intensively at the extraoral regions, especially at the lower lip (CDT, WDT), than in the intraoral regions, defining them as an evolutionary shield for food before it enters the mouth/body and causes harm [21]. Similar results were found for the lip mucosa [22]. Comparing the thermal detection thresholds of the chin and the lower lip with the cheek [2,4], cold is earlier detectable at the cheek, as well as it is earlier painful. The perception of warm is similar at the lower lip and the cheek, whereas the warm detection thresholds were higher for the chin and even more clearly for the tongue and gingiva. The lower lip was most sensitive for heat pain, while the intraoral mucosa (gingiva) was the least sensitive. For the tongue tip, the mechanical detection was like chin and lower lip, whereas for the gingiva the detection threshold was higher, as was the differentiation between pointed and blunt stimuli (MPT). At the gingiva, both touch perception (MDT) and point/blunt discrimination (MPT) were delayed. Therein founded the pointed stimuli (MPS) were not as painful at the gingiva than for the extraoral areas. Similar results were found for the gingival mucosa of the upper premolar region [18]. Contrary to the recommendation of Svensson et al. [10], we did not include measurements with pressure (PPT) in the examination protocol, because preliminary tests showed that it was not possible to apply pressure to the areas in a meaningful way due to the anatomical conditions. Therefore, pain is understandable, although sensitivity of the gingiva is lower compared to other parts of the face such as the tongue [9,18,22]. This is not according to Pigg et al. [18], who described PPT lower at the tongue than at the mucosal surface. VDT was not used due to anatomical conditions. Since Aß-fibers are already tested at MDT, testing vibration does not seem to provide any additional information. We found no significant differences for wind-up ratio (WUR) between the orofacial sites. 

No effect was seen concerning the QST parameters according to the age, except for thermal detection thresholds. Pigg et al. [18] also found no significant differences between younger (<40) and older (>40) subjects, whereas Rolke et al. [2,4] found higher thresholds for older subjects in thermal QST parameters. Significantly lower sensitivity for WDT and CDT were found in the older group compared to the younger group at the tongue tip and mucosal lip [22,23] and at the chin [24]. Sensitivity of the face seems not to be highly affected by an increasing age according to literature [22,23,24]. Age had no influence on pain thresholds in this study. This might be irritable because in medicine in general, a diminished pain threshold [25,26,27] is known to take place. A possible reason might be that with the facial area serving as a potential protection shield for us, sensory function should be unchanged. 

In this study, gender did not influence thermal and mechanical thresholds. Other studies report gender differences [27]. This might be because of different sample sizes or various hormone balances. Menstrual cycle and menopause may influence the results. Women were evaluated to exhibit lower pain thresholds in many QST tests [24]. This may be due to different contact areas of the thermode in men and women. As a consequence, the A-delta and C-fibers may differ with a consecutive different QST assessment [28,29]. Significant gender differences were found with less sensitivity for WDT and MDT in men compared to women [23], and a lower threshold for CDT and HPT in women [22]. However, an evaluation of sensory function of the face was performed in equine [30], which reported on increased thresholds (thermal and mechanical) according to age. Gender had no influence on the thresholds, nor does shaving the investigated area [30]. One might assume more sensitive skin sensation in women because of lower skin thickness, softertexture of the skin and more superficial vascularization and innervation. In general, studies report a lower pain level in women [31,32]. An additional finding could be documented comparing shaved or not shaved skin area in men [30]. This too had no effect on the sensory function in men in our study. One might speculate that a constant continuous mechanical trigger such as shaving might lead to a hypersensitization with potential hyperalgesia (or the opposite). This was not found in this study, which reported about equal thresholds for women and men. 

In conclusion, the face is the most sensitive region of the body [20]. On the face, the posterolateral region is not that sensitive as the median part. This shift of sensitivity was described as a reason of changing innervation density and different fiber ratios [33]. 

### 4.2. Taste

Because of topical interest, patients with a prolonged dysgeusia should be asked about their COVID-19 history. Apart from this, long-COVID-syndrome is known to be associated with neuropathic pain conditions and hypoalgesia. To enable diagnostics in this field, reference data are inevitable. 

Standard values for thermal and mechanical detection and pain thresholds have been obtained for the cheek and intraoral mucosa [34], but not the whole functional pattern including taste qualities. In this study, a significant influence of gender on the taste of salty, bitter and sour could be determined. Women seem to have a much better sense of taste concerning these taste qualities. This goes along with the results of the study by Landis et al. [35] Another study found a gender and age-decreasing taste sensibility in men [36]. The same was evaluated by Fikentscher 1977 [37]. In elderly men (>40), a decreased taste sensibility was found. Possible reasons for the better gustatory functions in women can be found in the hormone composition. During pregnancy, with a consecutive change of hormones, changes of the taste occur. A better sensitivity for “bitter” may protect the fetus against any intoxication. Therefore, hormones are known to influence the taste sensibility [38]. Even anatomy is gender dependent. Women have more fungiform papilla and taste buds than men [39]. 

Concerning age, taste sensibility is negatively affected in the taste qualities of sour and especially bitter [35]. This was supported by other studies reporting on taste loss associated with increasing age [40]. This may also be influenced by chronic disease and polypharmacy, which was excluded in this study at the beginning by defining the exclusion criteria. No influence was found for “sweet” [40]. This may lead to mal-nutrition and more consumption of sweet food, triggering further health-care problems. Consistent with previous studies [41,42], the sensation of ‘sweet’ was very easy to detect compared to the other tastes. Moreover, sweet taste is described as a “special taste quality”, pointing to addiction models eliciting neural responses similar to previous experiences with drugs [43].

Some subjects were not able to distinguish between sour and salty. This might result from the frequent combination of both tastes in daily food [42]. An additional part may be the evolutionary history of sour taste [43,44,45]. It might be assumed that country-dependent and cultural influence of the food might alternate the taste sensibility [46]. 

Taste strips were easy to apply (precise, small detection area) and well established for detecting taste sensibility. Only the corresponding taste buds were evaluated. In contrary to Landis et al. [35], who worked with the “forced choice” method, in our study, subjects classified indistinguishable taste qualities as “not noticeable” which ruled out false-positive answers. 

### 4.3. Limitations of the Study

We applied the QST protocol of the DFNS in the orofacial area and extended it to intraoral test sites by only slight adaptation of the stimuli. Good internal validity of this protocol has been shown [4,19]. We considered a typical sample of the population considering different ages and gender. Therefore, we are confident that external validity of our findings is high.

For daily practice, this quantitative sensory testing protocol might be too time consuming and sophisticated, implicating a limitation of the method [47]. 

Possible limitations of the study might also be seen in the missing measurement of PPT or VDT. One might assume these findings are necessary to evaluate the whole somatosensory profile. Due to anatomical conditions, pressure was avoided in these areas (lower lip, gingiva, tongue). The evoked pain might have distorted the measurements in sense of too much pain (false positive effect). Same goes for VDT. No additional information could be recruited since Aß-fibers are already tested at MDT. 

To excluded gender-specific results, measurements could have been performed only at one gender. This might have been unrealistic and would have not represent a correct point of view to general population. 

Concerning taste, one might criticize by noting that the taste quality “umami” was not taken into consideration. Yoshida and Ninomiya [48] described no possible differentiation between sweet and umami because of the same receptor subunits assessed in mice (T1R3). They corresponded with both tastes.

## 5. Conclusions

This study provides comprehensive reference data for the extra- and intraoral trigeminal region (V3) including gustatory testing. Based on such norm data, an early detection of sensory plus and minus signs such as hypoesthesia or hyperalgesia is possible. QST can therefore be applied as a useful diagnostic tool for early detection of orofacial diseases, such as diabetic polyneuropathy, chemotherapy-induced neuropathy, post-herpetic neuralgia or burning mouth syndrome. In addition, neurophysiological changes and/or pain due to peripheral nerve injury or associated tumor diseases can be more accurately classified for therapeutical treatment. QST not only has clinical and experimental relevance, but it can also be used as a method for the preparation of expert opinions. This neurophysiological profile can be supplemented by testing the taste sensitivity. Gender seems to play a significant influence on the perception of the taste of salty, bitter and sour. In general, women have a much better sense of taste.

Data of this quantitative sensory testing can serve as baseline for describing neurophysiological changes of the trigeminal nerve V3, e.g., in orofacial diseases or chronic pain states.

## Figures and Tables

**Table 1:** Orofacial sensory reference data. Paradoxical heat sensation (PHS) and dynamic mechanical allodynia (DMA) did not significantly occur in healthy subjects. (QST = Quantitative Sensory Testing, CDT = Cold Detection Threshold, CPT = Cold Pain Threshold, WDT = Warm Detection Threshold, HPT = Heat Pain Threshold, MDT = Mechanical Detection Threshold, MPS = Mechanical Pain Sensitivity, MPT = Mechanical Pain Threshold, TSL = Thermal Sensory Limen, WUR = Wind Up Ration, SEM = standard error of the mean).

		QST Data Male Subjects		QST Data Female Subjects	
QST Parameter	Orofacial Areal	<40 Years (*n* = 18)	>40 Years (*n* = 22)	<40 Years (*n* = 32)	>40 Years (*n* = 30)
		QST Mean Raw Data ± SEM or Retransformed Data *QST Log Data ± SEM*	QST Mean Raw Data ± SEM or Retransformed Data QST Log Data ± SEM	QST Mean Raw Data ± SEM or Retransformed Data QST Log Data ± SEM	QST Mean Raw Data ± SEM or Retransformed Data QST Log Data ± SEM
		(▼▲ 95% Confidence Interval)	(▼▲ 95% Confidence Interval)	(▼▲ 95% Confidence Interval)	(▼▲ 95% Confidence Interval)
**CDT (Δ°C)**	chin	**1.88** (▼1.37▲2.58)/*0.63 ± 0.16*	**2.71** (▼2.03▲3.6)/*1.0 ± 0.15*	**2.31** (▼1.82▲2.93)/*0.84 ± 0.12*	**2.50** (▼1.96▲3.2)/*0.92 ± 0.12*
	lower lip	**1.14** (▼0.85▲1.55)/*0.13 ± 0.15*	**2.13** (▼1.62▲2.8)/*0.76 ± 0.14*	**1.35** (▼1.08▲1.69)/*0.30 ± 0.11*	**1.4** (▼1.11▲1.77)/*0.34 ± 0.12*
	tongue	**2.23** (▼1.47▲3.38)/*0.80 ± 0.21*	**4.43** (▼3.04▲6.47)/*1.49 ± 0.19*	**3.45** (▼2.52▲4.72)/*1.24 ± 0.16*	**3.14** (▼2.27▲4.34)/*1.15 ± 0.16*
	gingiva	**1.95** (▼0.99▲3.85)/*0.67 ± 0.35*	**4.74** (▼2.56▲8.78)/*1.56 ± 0.31*	**2.29** (▼1.38▲3.82)/*0.83 ± 0.26*	**2.67** (▼1.57▲4.52)/*0.98 ± 0.27*
**WDT (Δ°C)**	chin	**5.56** (▼4.44▲6.94)/*1.71 ± 0.11*	**7.08** (▼5.77▲8.67)/*1.96 ± 0.10*	**6.22** (▼5.26▲7.36)/*1.83 ± 0.09*	**4.98** (▼4.19▲5.93)/*1.61 ± 0.09*
	lower lip	**1.47** (▼1.17▲1.84)/*0.39 ± 0.11*	**2.45** (▼2.01▲3.01)/*0.9 ± 0.10*	**1.66** (▼1.40▲1.96)/*0.50 ± 0.09*	**2.00** (▼1.68▲2.38)/*0.69 ± 0.09*
	tongue	**8.50** (▼6.8▲10.63)/*2.14 ± 0.11*	**8.83** (▼7.21▲10.81)/*2.18 ± 0.10*	**10.06** (▼8.50▲11.9)/*2.31 ± 0.09*	**6.91** (▼5.81▲8.22)/*1.93 ± 0.09*
	gingiva	**13.54** (▼10.82▲16.94)/*2.61 ± 0.11*	**16.2** (▼13.23▲19.83)/*2.8 ± 0.09*	**16.41** (▼13.87▲19.41)/*2.8 ± 0.10*	**15.78** (▼13.27▲18.77)/*2.76 ± 0.09*
**TSL (°C)**	chin	**7.16** (▼5.43▲9.44)/1.97 ± 0.14	**9.43** (▼7.34▲12.11)/*2.24 ± 0.13*	**6.86** (▼5.57▲8.44)/*1.93 ± 0.11*	**7.13** (▼5.75▲8.83)/*1.96 ± 0.14*
	lower lip	**2.59** (▼2.03▲3.30)/*0.95 ± 0.12*	**4.34** (▼3.48▲5.41)/*1.47 ± 0.11*	**2.87** (▼2.39▲3.45)/*1.05 ± 0.09*	**3.61** (▼2.99▲4.36)/*1.28 ± 0.1*
	tongue	**9.32** (▼7.52▲11.56)/*2.21 ± 0.09*	**13.50** (▼11.12▲16.41)/*2.38 ± 0.08*	**10.79** (▼9.18▲12.68)/*2.60 ± 0.1*	**9.10** (▼7.70▲10.75)/*2.23 ± 0.11*
	gingiva	**10.62** (▼8.2▲13.75)/*2.36 ± 0.13*	**15.72** (▼12.44▲29.85)/*2.76 ± 0.12*	**13.47** (▼11.1▲16.35)/*2.60 ± 0.1*	**13.43** (▼11▲16.41)/*2.6 ± 0.10*
**CPT(°C)**	chin	**15.45 ± 2.59** (▼10.34▲20.56)	**11.11 ± 2.35** (▼6.5▲15.74)	**15.65 ± 1.95**(▼11.82▲19.49)	**14.84 ± 2.01** (▼10.88▲18.80)
	lower lip	**11.54 ± 2.9** (▼5.82▲17.26)	**19.21 ± 2.62** (▼14.04▲24.38)	**15.1 ± 2.18** (▼10.81▲19.39)	**16.84 ± 2.25** (▼12.41▲21.26)
	tongue	**16.09 ± 2.69** (▼10.8▲21.39)	**13.98 ± 2.43** (▼9.19▲18.77)	**13.6 ± 2.01** (▼9.63▲17.57)	**16.54 ± 2.08** (▼12.44▲20.65)
	gingiva	**21.58 ± 1.34** (▼18.94▲24.22)	**21.63 ± 1.21** (▼19.24▲24.02)	**20.66 ± 1.00** (▼18.68▲22.64)	**22.47 ± 1.04** (▼20.43▲24.52)
**HPT (°C)**	chin	**44.61 ± 1.05** (▼42.54▲46.68)	**47.75 ± 0.95** (▼45.87▲49.62)	**45.17 ± 0.79** (▼43.62▲46.73)	**45.44 ± 0.81** (▼43.84▲47.05)
	lower lip	**45.29 ± 1.25** (▼42.82▲47.75)	**42.36 ± 1.13** (▼40.13▲44.59)	**44.53 ± 0.94** (▼42.68▲46.38)	**42.13 ± 0.97** (▼40.22▲44.04)
	tongue	**47.19 ± 1.12** (▼44.99▲49.39)	**46.47 ± 1.01** (▼44.48▲48.46)	**47.37 ± 0.84** (▼45.72▲49.02)	**45.66 ± 0.87** (▼43.95▲47.36)
	gingiva	**48.16 ± 0.58** (▼47.02▲49.30)	**49.5 ± 0.52** (▼48.47▲50.53)	**49.02 ± 0.43** (▼48.17▲49.88)	**48.83 ± 0.45** (▼47.95▲49.71)
**MDT (mN)**	chin	**0.06** (▼0.04▲0.08)/*−2.87 ± 0.16*	**0.06** (▼0.05▲0.08)/*−2.79 ± 0.15*	**0.05** (▼0.04▲0.06)/*−3.05 ± 0.12*	**0.04** (▼0.03▲0.06)/*−3.13 ± 0.12*
	lower lip	**0.07** (▼0.06▲0.09)/*−2.63 ± 0.13*	**0.1** (▼0.08▲0.12)/*−2.34 ± 1.12*	**0.05** (▼0.04▲0.06)/*−2.94 ± 0.1*	**0.06** (▼0.05▲0.07)/*−2.90 ± 0.10*
	tongue	**0.05** (▼0.04▲0.07)/*−3.01 ± 0.16*	**0.07** (▼0.06▲0.1)/*−2.6 ± 0.14*	**0.06** (▼0.05▲0.08)/*−2.78 ± 0.12*	**0.05** (▼0.04▲0.07)/*−2.06 ± 0.12*
	gingiva	**3.89** (▼1.4 ▲10.79)/*1.36 ± 0.52*	**3.41** (▼1.35▲8.6)/*1.23 ± 0.47*	**4.39** (▼2.04▲9.44)/*1.48 ± 0.39*	**4.37** (▼1.98▲9.63)/*1.47 ± 0.40*
**MPT (mN)**	chin	**11.76** (▼7.1 ▲19.48)/*2.46 ± 0.26*	**10.04** (▼6.36▲15.85)/*2.31 ± 0.23*	**16.93** (▼11.59▲24.72)/*2.83 ± 0.19*	**10.90** (▼7.37▲16.12)/*2.39 ± 0.2*
	lower lip	**9.89** (▼6.88▲14.2)/*2.29 ± 0.18*	**8.18** (▼5.9 ▲11.35)/*2.10 ± 0.17*	**9.35** (▼7.13▲12.27)/*2.24 ± 0.14*	**11.91** (▼8.99▲15.76)/*2.48 ± 0.14*
	tongue	**17.62** (▼10.5 ▲29.56)/*2.87 ± 0.26*	**13.76** (▼8.61▲21.97)/*2.62 ± 0.24*	**24.36** (▼16.52▲35.91)/*3.19 ± 0.2*	**23.21** (▼13.55▲34.66)/*3.15 ± 0.20*
	gingiva	**17.96** (▼9.86▲32.70)/*2.89 ± 0.30*	**21.79** (▼12.67▲37.47)/*3.08 ± 0.28*	**13.34** (▼8.51▲20.91)/*2.59 ± 0.23*	**21.86** (▼13.74▲34.77)/*3.09 ± 0.24*
**MPS (0–100)**	chin	**0.86** (▼0.34▲2.00)/*−0.16 ± 0.43*	**1.07** (▼0.5 ▲2.29)/*0.06 ± 0.39*	**1.19** (▼0.63▲2.25)/*0.178 ± 0.32*	**1.73** (▼0.9 ▲3.33)/*0.55 ± 0.33*
	lower lip	**0.97** (▼0.47▲2.04)/*−0.03 ± 0.37*	**0.68** (▼0.35▲1.32)/*−0.39 ± 0.34*	**1.24** (▼0.71▲2.16)/*0.22 ± 0.28*	**1.19** (▼0.67▲2.12)/*0.178 ± 0.29*
	tongue	**0.57** (▼0.27▲1.23)/*−0.56 ± 0.39*	**0.59** (▼0.3 ▲1.17)/*−0.53 ± 0.35*	**0.45** (▼0.25▲0.79)/*−0.81 ± 0.29*	**0.8** (▼0.44▲1.44)/*−0.23 ± 0.3*
	gingiva	**0.58** (▼0.27▲1.25)/*−0.54 ± 0.39*	**0.33** (▼0.17▲0.67)/*−1.10 ± 0.35*	**0.51** (▼0.28▲0.90)/*−0.68 ± 0.29*	**0.68** (▼0.38▲1.24)/*−0.38 ± 0.30*
**WUR (0–100)**	chin	**2.43** (▼1.62▲3.65)/*0.89 ± 0.21*	**2.45** (▼1.69▲3.54)/*0.9 ± 0.19*	**2.59** (▼1.89▲3.55)/*0.95 ± 0.16*	**2.57** (▼1.87▲3.52)/*0.94 ± 0.16*
	lower lip	**2.10** (▼1.43▲3.09)/*0.74 ± 0.2*	**3.09** (▼2.18▲4.38)/*1.13 ± 0.18*	**2.27** (▼1.70▲3.03)/*0.82 ± 0.15*	**2.61** (▼1.94▲3.52)/*0.96 ± 0.15*
	tongue	**3.22** (▼2.18▲4.77)/*1.17 ± 0.2*	**3.47** (▼2.44▲4.95)/*1.25 ± 0.18*	**2.54** (▼1.9 ▲3.41)/*0.93 ± 0.15*	**3.21** (▼2.37▲4.35)/*1.17 ± 0.15*
	gingiva	**3.12** (▼1.99▲4.88)/*1.14 ± 0.23*	**2.45** (▼1.63▲3.68)/*0.9 ± 0.21*	**3.76** (▼2.65▲5.32)/*1.32 ± 0.18*	**3.04** (▼2.15▲4.31)/*1.11 ± 0.18*

**Table 2:** Analysis of side effects for orofacial reference data (*p*-values/Student’s *t*-test). (CDT = Cold Detection Threshold, WDT = Warm Detection Threshold, TSL = Thermal Sensory Limen, CPT = Cold Pain Threshold, HPT = Heat Pain Threshold, MDT = Mechanical Detection Threshold, MPT = Mechanical Pain Threshold, MPS = Mechanical Pain Sensitivity, WUR = Wind Up Ration). * *p* < 0.05; ** *p* < 0.001.

	Chin	Lower Lip	Gingiva	Tongue
**CDT**	0.886	0.85	0.422	0.757
**WDT**	0.903	0.003 **	0.63	0.107
**TSL**	0.521	0.103	0.431	0.32
**CPT**	0.08	0.654	0.192	0.796
**HPT**	0.641	0.054	0.658	0.31
**MDT**	0.044 *	0.865	0.649	0.865
**MPT**	0.526	0.567	0.553	0.572
**MPS**	0.101	0.690	0.386	0.384
**WUR**	0.585	0.735	0.643	0.65

**Table 3:** Analysis of variance of orofacial reference data. (CDT = Cold Detection Threshold, WDT = Warm Detection Threshold, TSL = Thermal Sensory Limen, CPT = Cold Pain Threshold, HPT = Heat Pain Threshold, MDT = Mechanical Detection Threshold, MPT = Mechanical Pain Threshold, MPS = Mechanical Pain Sensitivity, WUR = Wind Up Ration). * *p* < 0.05; ** *p* < 0.001; *** *p* < 0.0001.

	Position			Age			Sex			Position ∗ Age			Position ∗ Sex			Age ∗ Sex			Position ∗ Age ∗ Sex		
	DF	F	*p*	DF	F	*p*	DF	F	*p*	DF	F	*p*	DF	F	*p*	DF	F	*p*	DF	F	*p*
**CDT**	3	19.04	<0.0001 ***	1	11.82	<0.001 ***	1	0.32	**0.574**	3	0.31	**0.818**	3	0.51	**0.674**	1	9.01	0.003 **	3	0.48	**0.696**
**WDT**	3	458.94	<0.0001 ***	1	0.98	**0.323**	1	0.19	**0.662**	3	6.71	0.002 **	3	1.01	**0.388**	1	7.08	0.008 *	3	0.42	**0.736**
**TSL**	3	129.82	<0.0001 ***	1	14.25	0.002 **	1	1.71	**0.193**	3	1.29	**0.278**	3	0.64	**0.589**	1	11.20	0.001 **	3	0.42	**0.738**
**CPT**	3	18.79	<0.0001 ***	1	0.67	**0.413**	1	0.36	**0.548**	3	1.58	**0.195**	3	0.22	**0.882**	1	0.27	**0.604**	3	1.00	**0.392**
**HPT**	3	33.63	<0.0001 ***	1	0.82	**0.368**	1	0.79	**0.375**	3	4.14	0.007 **	3	0.33	**0.804**	1	1.86	**0.174**	3	0.5	**0.680**
**MDT**	3	117.27	<0.0001 ***	1	0.19	**0.666**	1	1.33	**0.249**	3	0.33	**0.802**	3	1.85	**0.139**	1	0.77	**0.381**	3	0.56	**0.644**
**MPT**	3	10.86	<0.0001 ***	1	0.3	**0.861**	1	2.24	**0.136**	3	1.3	**0.275**	3	0.92	**0.431**	1	0.54	**0.462**	3	0.58	**0.626**
**MPS**	3	5.42	0.0013 **	1	0.15	**0.698**	1	2.74	**0.1**	3	0.63	**0.504**	3	0.28	**0.84**	1	1.91	**0.169**	3	0.19	**0.902**
**WUR**	3	1.82	**0.145**	1	0.28	**0.6**	1	0.02	**0.880**	3	1.30	**0.275**	3	0.69	**0.6**	1	0.01	**0.921**	3	0.25	**0.864**

## Data Availability

The data presented in this study are available on request from the corresponding author. The data are not publicly available due to ethical reasons.
